# Congenital diaphragmatic hernia: Misdiagnosis in adolescence

**DOI:** 10.4103/0971-9261.44775

**Published:** 2009

**Authors:** Yogender Singh Kadian, Kamal Nain Rattan, Manish Verma, Pradep Kajal

**Affiliations:** Department of Pediatric Surgery, Pt.B.D. Sharma P.G.I.M.S. Rohtak, India

**Keywords:** Congenital diaphragmatic hernia, gastric injury, tuberculosis

## Abstract

We report 3 cases of congenital diaphragmatic hernia (CDH) in the second decade of life which were misdiagnosed on initial presentation. The first case had an iatrogenic gastric injury because of intercostal tube drainage for suspected pleural effusion. The second case was treated for pulmonary tuberculosis for 6 months before being diagnosed as a case of CDH. The third case presented as acute chest pain on the left side. It was treated accordingly for 1 month and was diagnosed as a CDH on a CT scan of the chest when seen by a surgeon.

## INTRODUCTION

Congenital diaphragmatic hernia generally presents in the first few hours of life and is even diagnosed antenataly. In later age groups misdiagnosis is a distinct possibility, with the risk of serious morbidity and mortality.[1,2] Here is a brief presentation of 3 cases of congenital diaphragmatic hernia presenting in the adolescent age group.

## CASE REPORTS

### Case 1

**Figure 1:**
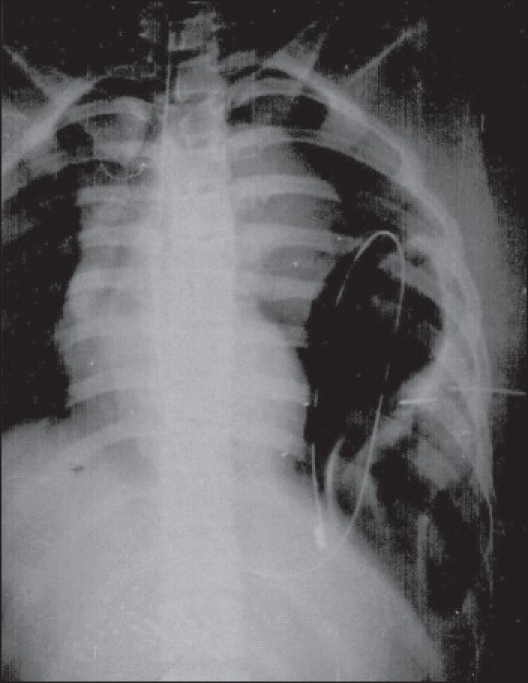
X-ray chest showing intercostal tube and Ryle's tube lying in the left hemithorax

A 12-year-old female was referred with intercostal tube drain in-situ on left side of her chest with bile coming out of the drain. The intercostal tube had been put in by a private practitioner to drain the suspected pleural effusion that actually was a diaphragmatic hernia with the stomach and small intestine in the thoracic cavity [Figure 1]. An exploratory laparotomy was done through a midline incision. There was a Bochdalek diaphragmatic defect on the left hemi- diaphragm and a hernial sac containing stomach and small gut in the chest cavity. A perforation in the anterior wall of the stomach was present, which was closed at surgery. The entire contents were reduced and the diaphragmatic defect was repaired. The postoperative period was uneventful except for a minor wound infection.

### Case 2

**Figure 2:**
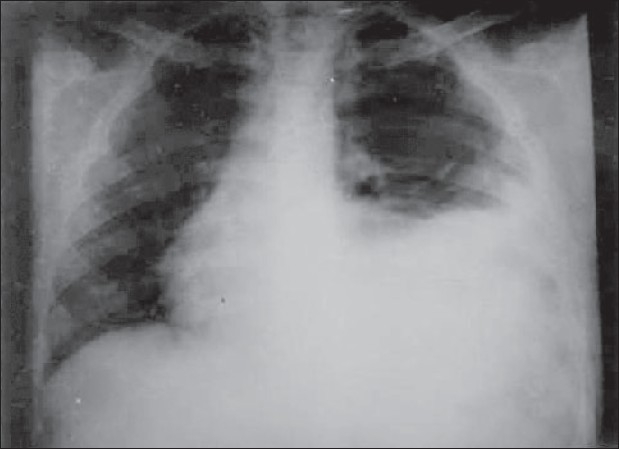
Chest radiograph on which child was treated for pulmonary tuberculosis

A 13-year-old male was being treated by a private practitioner for recurrent chest infections. He also had received anti-tubercular treatment for 6 months on trial basis with mild relief of symptoms. He was referred to our institute. On an X-ray of his chest, the left hemi-diaphragm was not clearly visible and gastric fluid level was absent [Figure 2]. A barium meal follow- through was done that showed stomach and small gut in the chest cavity. An exploratory laparotomy through a midline incision revealed a typical Bochdalek hernia with stomach, small gut, and a portion of colon and spleen in the left chest cavity. The contents were reduced into the abdominal cavity and a splenectomy was done as the spleen was ruptured during its reposition back into the abdomen. The postoperative recovery was uneventful.

### Case 3

A 14-year-old male was admitted to the hospital with a history of acute pain in the left side of his chest and dyspepsia for 4 days. The pain was a constant, dull aching and more upon deep inspiration. The pain was severe and associated with vomiting on the day of admission. There was decreased air entry at the left posterior side of the chest, but no other abnormality on auscultation. The abdomen was scaphoid but non- tender. A diagnosis of late onset of a left congenital diaphragmatic hernia was made after a computed tomography (CT) scan of the chest and was operated on by abdominal approach. During surgery, the stomach, small gut, colon, and spleen were lying in the left thoracic cavity. Reduction of contents and repair of defect was done with 2-0 non-absorbable sutures. Three months after the operation, the patient started having respiratory distress and an X-ray of the chest was taken that showed gut loops in the left thoracic cavity (recurrence of hernia). The patient was again operated on and a manual reduction of contents was performed. The defect was repaired after adequately mobilizing the posterior leaf of the diaphragm and double breasting of the diaphragm was done. A chest tube was put in on the left side and the patient was discharged 7 days after the operation. The patient was well at a 6 month follow-up visit.

## DISCUSSION

Most cases of congenital diaphragmatic hernia are diagnosed within the first few hours of life, with 5 to 25% of diaphragmatic hernias appearing beyond the neonatal period, with age at discovery from 1 month to late adulthood.[3] Delayed presentation in late childhood or adolescence poses a dilemma in diagnosis as most of these cases are managed by general surgeons with less experience in the clinical entity. Haines and Collins reported an asymptomatic adult diagnosed with a diaphragmatic hernia after a chest radiograph was interpreted as showing a left pleural effusion that layered in the left lateral decubitus position.[4] Hegarty, *et al*. have reported 2 cases where intercostal drainage of gastric contents provided a diagnosis of diaphragmatic hernia.[5] A diaphragmatic hernia should be suspected even after a trivial trauma if an erect plain radiograph of the chest shows an absence of fundic bubble in its normal position. A high index of suspicion is required. Simple investigations like a chest radiograph can show air or fluid filled loops in the left hemithorax and a shift of cardiac silhouette to the right. Moreover, if a nasogastric tube is placed before a chest radiograph, it can delineate the position of the intrathoracic stomach. Ultrasonography is useful in the diagnosis of congenital diaphragmatic hernia where uninterrupted contours of the diaphragm are not seen and peristalsis of the bowel can be observed in the thorax.[6,7] Contrast studies and CT scans of the thoracic and abdominal cavity are specific in making the diagnosis, but are not always necessary for making a diagnosis in every case. A CT scan may not be able to directly image the diaphragmatic lacerations that lie in different scan planes.[8] Treatment of the condition involves repositioning of abdominal contents into the abdominal cavity and closure of the diaphragmatic defect preferably by the abdominal route.

To conclude, congenital diaphragmatic hernia presenting in adolescence is rare and shows non specific symptoms. Many of these patients may require urgent treatment to avoid complications. Hence, a child presenting with recurrent gastrointestinal or respiratory complaints should be assessed thoroughly and a high index of suspicion is required to successfully diagnose and manage this condition in the adolescent age group.

## References

[1] Berman L, Stringer D, Ein S, Shandling B (1988). The late-presenting pediatric Bochdalek hernia: A 20-year review. J Pediatr Surg.

[2] Newman BM, Afshani E, Karp MP, Jewett TC, Cooney DR (1986). Presentation of congenital diaphragmatic hernia past the neonatal period. Arch Surg.

[3] Mishalany H, Gordo J (1986). Congenital diaphragmatic hernia in monozygotic twins. J Pediatr Surg.

[4] Haines JO, Collins RB (1970). Bochdalek hernia in an adult simulating a pleural effusion. Radiology.

[5] Hegarty MM, Bryer JV, Angorn IB, Baker LW (1978). Delayed presentation of traumatic diaphragmatic hernia. Ann Surg.

[6] Wooldridge JL, Partrick DA, Bensard DD, Deterding RR (2003). Diaphragmatic hernia simulating a left pleural effusion. Pediatrics.

[7] Benacerraf BR, Greene MF (1986). Congenital diaphragmatic hernia: US diagnosis prior to 22 weeks of gestation. Radiology.

[8] Heiberg E, Wolverson MK, Hurd RN, Jagannadharao B, Sundaram M (1980). CT recognition of traumatic rupture of diaphragm. AJR Am J Roentgenol.

